# Gut microbiota heterogeneity in non-alcoholic fatty liver disease: a narrative review of drivers, mechanisms, and clinical relevance

**DOI:** 10.3389/fmicb.2025.1645298

**Published:** 2025-10-15

**Authors:** Ying Guo, Naisi Zhang, Dongmei Pei

**Affiliations:** Department of Health Management, Shengjing Hospital of China Medical University, Shenyang, China

**Keywords:** gut microbiota, non-alcoholic fatty liver disease, metabolic syndrome, confounding variables, population-based cohort studies

## Abstract

Non-alcoholic fatty liver disease (NAFLD), a prevalent metabolic disorder, is increasingly recognized as a complex condition influenced by gut microbiota dysbiosis. However, the heterogeneity in findings across studies has hindered the clinical translation of microbiota-based interventions. In this narrative review, we synthesize current evidence on gut microbial alterations in patients with NAFLD, with a focus on the sources of variability that contribute to inconsistent results. We included human studies (2000–2024) that compared gut microbiota profiles between NAFLD patients and healthy controls using 16S rRNA or metagenomic sequencing; key drivers of microbial changes include clinical factors (metabolic comorbidities, disease progression), biological variables (diet, genetics), and methodological biases (sequencing platform differences, diagnostic criteria variability). Emerging evidence highlights the role of non-bacterial components (fungi, viruses) in modulating bacterial communities and disrupting host metabolic pathways, exacerbating hepatic inflammation and lipid accumulation. To overcome current limitations, we propose integrating multi-omics approaches (metagenomics, metabolomics, and proteomics) with a longitudinal study design to capture dynamic microbiota–host interactions. Precision microbiota therapies, including strain-specific probiotics, engineered microbial consortia, and fecal microbiota transplantation tailored to individual dysbiosis profiles, are emerging as promising strategies for targeted interventions. Addressing these challenges is essential to identifying reliable microbial biomarkers and developing personalized strategies for NAFLD prevention and treatment. Future research should harmonize methodologies, validate causal mechanisms, and optimize microbiota-based therapies to bridge experimental findings and clinical application.

## 1 Introduction

Advances in the gut–liver axis (GLA) theory have highlighted the pathophysiological association between gut microbiota dysbiosis and non-alcoholic fatty liver disease (NAFLD), making it a key research focus in metabolic liver disease (Tilg et al., 2022). Evidence suggests that structural disruptions, metabolite imbalances, and intestinal barrier dysfunction in the gut microbiota are not only correlated with dietary patterns and NAFLD progression but also involved in exacerbating hepatic steatosis and inflammatory responses through microbe-host interactions (Ni et al., 2023).

High-throughput sequencing technologies have enabled researchers to conduct large cohort studies, revealing novel gut microbial profiles in patients with NAFLD (Sookoian et al., 2024). However, inconsistent findings have hindered consensus on NAFLD-associated microbial signatures. At the taxonomic level, differences between obese and non-obese patients remain unresolved, particularly regarding functional genomics and metabolomics (Yan et al., 2024; Gagnon et al., 2023). Furthermore, inconsistent findings regarding alpha diversity indices (e.g., Shannon index) often arise from cohort heterogeneity and differences in disease staging (e.g., the distinction between simple steatosis and NASH). Moreover, the confounding effects of prevalent metabolic comorbidities further complicate the interpretation of these metrics, leading to non-comparable results across studies (Ng et al., 2025).

**Figure 1:**
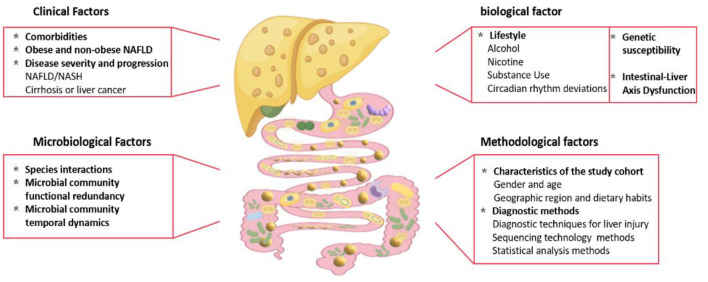
Factors contributing to heterogeneity in gut microbiota characteristics of NAFLD patients. The heterogeneity in gut microbiota composition among NAFLD patients is driven by a multifactorial interplay of clinical, biological, methodological, and microbial dynamics (Fig. 1). Key contributors: 1. Clinical factors: Overlapping microbial signatures due to comorbid metabolic disorders (e.g., obesity, insulin resistance, type 2 diabetes). Distinct taxonomic profiles between obese and non-obese NAFLD subphenotypes. 2. Biological drivers: Dietary patterns (e.g., high-fat diets, low fiber intake). Genetic predisposition (e.g., PNPLA3 polymorphisms). Gut-liver axis (GLA) dysfunction, including intestinal barrier disruption and systemic inflammation. 3. Methodological limitations: Cohort heterogeneity (geographical, ethnic, and lifestyle disparities). Technical variability (16S rRNA vs. shotgun sequencing, bioinformatics pipelines). Insufficient sample sizes and inconsistent disease classification criteria. 4. Microbial community dynamics: Functional redundancy among microbial taxa. Competitive/synergistic species interactions (e.g., Bacteroides vs. Prevotella dominance). Transient shifts in microbial abundance were influenced by host-derived metabolites.

Approximately 40% of the patients with NAFLD have comorbid conditions such as obesity, insulin resistance, or type 2 diabetes (T2DM), yet many studies fail to control for these overlapping conditions, leading to non-comparable results (Kim et al., 2024; Hov and Karlsen, 2023). The heterogeneity in the gut microbiota among patients with NAFLD arises from multiple sources (Figure 1), including clinical factors such as overlapping microbial profiles due to metabolic comorbidities, distinctions between obese and non-obese patients with NAFLD, and variation across disease stages (e.g., simple steatosis vs. NASH); biological contributors such as diet, genetics, and GLA dysfunction; methodological factors such as cohort differences, sequencing methods, analytical tools, and small sample sizes; and microbial dynamics such as species interactions and functional redundancy further complicate interpretations.

Critically, neglecting these sources of heterogeneity not only obscures genuine microbial signatures of NAFLD but also hinders the development of effective microbiota-based diagnostics and therapeutics. A nuanced understanding of these drivers is, therefore, a prerequisite for translating microbiome research into personalized clinical strategies. Against this backdrop, this review synthesizes current evidence on gut microbiota alterations in NAFLD, focusing on (1) microbial changes across disease phenotypes and progression stages, and (2) drivers of heterogeneity, including clinical confounders, biological variables, and methodological biases. By critically appraising these factors, we aim to bridge translational gaps and inform personalized NAFLD therapies, and advocate for multi-omics studies and standardized protocols.

## 2 Methods

This narrative review aimed to synthesize and critically evaluate the existing literature on the heterogeneity of gut microbiota in NAFLD. To ensure comprehensive coverage of the topic, we employed a systematic search strategy, although this review does not constitute a formal systematic review or meta-analysis requiring adherence to the Preferred Reporting Items for Systematic reviews and Meta-Analyses guidelines.

We systematically searched PubMed, Google Scholar, and Web of Science for studies published between January 1, 2000, and December 31, 2024. Search terms included “non-alcoholic fatty liver disease”, “NAFLD”, “gut microbiota”, “microbiome”, “heterogeneity”, and “metabolic syndrome”. Reference lists of relevant articles were manually searched to identify additional studies.

The inclusion criteria were (1) human studies involving patients with NAFLD; (2) gut microbiota analysis (e.g., 16S rRNA sequencing or metagenomic sequencing); (3) reported differences in microbiota between patients with NAFLD and healthy controls; and (4) sufficient data for analysis. Animal experiments, reviews, and case reports were excluded. Two investigators independently screened studies and extracted data on study design, sample size, participant characteristics, sequencing methods, and key findings. Disagreements were resolved through discussion.

The quality and relevance of the included studies were appraised by the authors in light of the study design, sample size, methodological consistency, and contribution to understanding the sources of heterogeneity. The synthesized evidence was then organized thematically around the key drivers of heterogeneity—clinical factors (e.g., comorbidities, disease severity), biological factors (e.g., diet, genetics), methodological factors (e.g., diagnostic criteria, sequencing techniques), and microbial community dynamics—to provide a critical analysis of the challenges and future directions in the field.

## 3 Factors affecting the heterogeneity of results of NAFLD gut microbiota studies

### 3.1 Clinical factors affecting heterogeneity of the results of NAFLD gut microbiota studies

#### 3.1.1 Comorbidities

NAFLD frequently co-occurs with a spectrum of metabolic comorbidities, including type 2 diabetes (T2DM), obesity, insulin resistance, dyslipidaemia, and hypertension. These conditions form a network with shared pathophysiological mechanisms and overlapping gut microbiota features (Quek et al., 2023). This overlap complicates the identification of NAFLD-specific microbial changes (Jarvis et al., 2020). For example, T2DM is associated with increased abundance of lipopolysaccharide (LPS)-producing genera (e.g., *Enterobacter*) and reduction in butyric acid-producing bacteria (e.g., *Faecalibacterium*); this pattern is also seen in patients with NAFLD, making it difficult to isolate microbiota signatures unique to NAFLD (Ojo et al., 2021; Yan et al., 2022). The synergistic role of T2DM in accelerating NAFLD progression was reported in large multicentre cohort studies, where patients with T2DM and NAFLD experienced faster progression of hepatic fibrosis and showed a higher risk of severe hepatic events than their non-T2DM counterparts (Huang et al., 2023b; Jarvis et al., 2020).

The Swedish Malmö cohort study (*n* = 12,548) identified low high-density lipoprotein cholesterol (< 1.0 mmol/L) and high triglycerides (TG≥1.7 mmol/L) levels as independent risk factors for severe hepatic events, with hazard ratios of 1.28 and 1.30, respectively (Nderitu et al., 2017). These metabolic disturbances may partly act through the gut microbiota (Li et al., 2023). For instance, Bifidobacterium lactis BL-99 supplementation improves lipid profiles and modulates bile acid metabolism by increasing the production of short-chain fatty acids (SCFAs) (Bajaj et al., 2018; Manaer et al., 2024).

Most NAFLD microbiome studies have insufficiently controlled for metabolic comorbidities. Few studies have adjusted for T2DM, and even fewer have accounted for other metabolic factors, such as dyslipidaemia (Loomba et al., 2019). This oversight can result in the misclassification of microbial markers, such as incorrectly attributing T2DM-related depletion in *Akkermansia* to NAFLD (Wong et al., 2013; Sanna et al., 2019; Dao et al., 2016).

Most previous studies have had a cross-sectional design, making it difficult to determine whether the microbial changes are a driver of NAFLD or a secondary outcome of metabolic comorbidities. Additionally, the majority of available cohort studies have focused on middle-aged and elderly populations, limiting the generalizability of the results. Future investigations should prioritize the implementation of large, cross-regional study cohorts to encompass a wider range of patients with NAFLD, particularly those with multiple comorbid metabolic disorders (Neeland et al., 2024) as well as adopt large, geographically diverse cohorts encompassing a broader demographic and comorbidity spectrum (Prado et al., 2024).

Personalized microbiota-based interventions, such as tailored probiotic or prebiotic blends (e.g., *Bifidobacterium animalis* subsp. *lactis* BL-99) for patients with T2DM-NAFLD, could improve lipid profiles and inhibit the progression of liver fibrosis. These strategies should be validated through randomized controlled trials to confirm their efficacy and safety.

#### 3.1.2 Obese and non-obese NAFLD

Obesity is a major risk factor for NAFLD; however, approximately 20% of the patients are lean, highlighting the phenotypic heterogeneity that complicates the elucidation of gut microbiota characteristics. Obesity plays a central role in driving disease heterogeneity through a distinct ‘lipid-flora-liver axis' mechanism (Nogacka et al., 2021; Leong et al., 2020; Zou et al., 2020; Chambers et al., 2019; Depommier et al., 2019; Mokkala et al., 2021). Gut microbiota profiles differ between lean and patients with obesity and NAFLD. Lean individuals exhibit a 1.5–2.3-fold increase in the abundance of *Akkermansia muciniphila* and depletion of *Firmicutes/Bacteroidetes* ratio (*p* < 0.05) (Kivimäki et al., 2022; Raman et al., 2013). In contrast, patients with obesity often show an increase in the relative abundance of LPS-producing bacteria genera, such as *Enterobacter*, and a depletion of butyric acid-producing bacteria, such as *Faecalibacterium prausnitzii* (Mocanu et al., 2021; Ghorbani et al., 2023; Zhang Z. et al., 2024). Of note, the abundance of *Firmicutes/Bacteroidetes* ratio peaks at a BMI of 33 kg/m^2^ and then declines (Haro et al., 2016). The non-linear relationship varies by sex; for instance, *Mycobacterium avium* decreases significantly with increasing BMI in men but not women (Kasai et al., 2015).

Bariatric surgery studies offer further insights into microbiota-host interactions. Postoperative increment in *A. muciniphila* (2.1–3.4-fold) is correlated with reduced hepatic adiposity (*r* = −0.47, *p* = 0.002) (Jian et al., 2022). However, higher baseline microbiota alpha diversity is associated with reduced postoperative fat loss (*p* = 0.016), indicating that diversity does not always confer metabolic benefits (Foster et al., 2017). This complexity reflects the influence of obesity-related factors such as insulin resistance and inflammation on microbiota function (Li et al., 2021). Moreover, Dong et al. (2023) reported that sleeve gastrectomy resulted in reduced glucose-dependent insulinotropic polypeptide signaling, conferring resistance to NAFLD. This effect was linked to elevated levels of *A. muciniphila* and changes in indolepropionic acid, a bacterial tryptophan metabolite. Bariatric surgery also alters ileal physiology, affecting bile acid composition independently of weight loss (Talavera-Urquijo et al., 2020).

Many previous studies have failed to adequately control for obesity-related confounders. BMI is a unidimensional indicator and may mask the effects of body fat distribution on the microbiome. Visceral fat area, for example, shows a stronger correlation with LPS-producing species than BMI (Nie et al., 2020; Yan et al., 2021). Future studies should incorporate precise indicators of body fat distribution and combine multidimensional data, such as computed tomography-measured visceral fat and metabolic markers (e.g., homeostasis model assessment of insulin resistance), to better define obesity-related microbiome changes in NAFLD.

Most microbiome studies have focused on bacteria, overlooking fungi and phages. Longitudinal studies are needed to assess microbiome remodeling after weight loss surgeries or interventions in different obesity phenotypes, emphasizing key taxa such as *A. muciniphila*. Additionally, the sex hormone-microbiota-hepatic steatosis regulatory axis should be examined separately for male and female patients with obesity to identify sex-specific therapeutic targets.

#### 3.1.3 Severity of NAFLD

The progression of NAFLD, from simple steatosis to NASH to liver fibrosis, cirrhosis, and hepatocellular carcinoma (HCC), significantly alters gut microbiota composition and function (Ponziani et al., 2019). Studies have shown that NAFLD/NASH is associated with an increased abundance of *Firmicutes/Bacteroidetes* ratio (1.5–2.3-fold) and reduced microbiome diversity (Shannon's index, decrease of 0.8–1.2, *p* < 0.05), along with shifts in key functional genera (Boursier et al., 2016; Vallianou et al., 2021).

In the Rotterdam cohort study, 472 out of 1,355 participants were diagnosed with hepatic steatosis, and their microbiotas were characterized by elevated levels of *Coprococcus* (fecal cocci) and *Ruminococcus*, with reduced microbial diversity (Alferink et al., 2021). Zhang et al. (2021) reported that a high-cholesterol diet promoted NASH progression, with an increase in *Mucispirillum, Desulfovibrio*, and *Anaerotruncus* and depletion of *Bifidobacterium* and *Bacteroides*. Hoyles et al. (2018) reported an increase in *Acidaminococcus, Escherichia coli*, and *Bacteroides* and depletion of *Faecalibacterium* and *Anaerobacterium* in patients with NASH.

**Figure 2:**
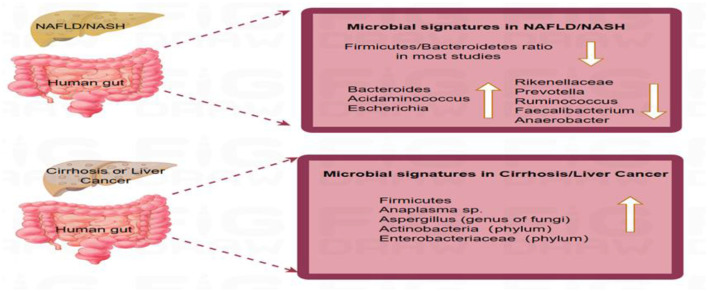
Distinct gut microbial profiles across the progression of non-alcoholic fatty liver disease. This schematic summarizes key microbial taxa whose relative abundance is consistently reported to be altered in NAFLD. The directional changes (increase ↑ or decrease ↓) depicted are based on a qualitative synthesis of the literature where a consistent change (e.g., >1.5-fold difference) was reported across multiple studies (e.g., Boursier et al., 2016; Ponziani et al., 2019, for bacteria; Demir et al., 2022, for fungi; Lang et al., 2020, for viruses). A. Microbial signatures in NAFLD/NASH: The Firmicutes/Bacteroidetes ratio is frequently altered (reduced in most studies). Commonly increased taxa include *Bacteroides* (genus), *Acidaminococcus* (genus), and *Escherichia* (genus). Commonly decreased taxa include Rikenellaceae (family), *Prevotella* (genus), *Ruminococcus* (genus), *Faecalibacterium* (genus), and *Anaerobacter* (genus). B. Microbial signatures in cirrhosis or hepatocellular carcinoma (HCC): Commonly increased taxa include phyla Firmicutes and Actinobacteria; family Enterobacteriaceae; genus *Anaeroplasma*. The dynamic divergence in microbial composition across disease stages underscores the gut microbiome's role in driving hepatic inflammation and fibrosis.

Shifts in the fungal profile also mark disease progression. A study of 69 patients with NAFLD showed that non-obese patients had a significantly increased proportion of *Trichoderma*/*Saccharomyces cerevisiae* (wood mold/sterile yeasts). This feature holds potential value for distinguishing between mild and advanced liver disease (Demir et al., 2022). As NAFLD progresses to cirrhosis and HCC, microbiota profiles evolve. Loomba et al. (2021) reported the predominance of *Firmicutes* in patients with NAFLD/NASH, and an increase in *Tenericutes* abundance in patients with advanced fibrosis. Ponziani et al. (2019) reported increments in *Enterobacteriaceae* and *Streptococcus* spp. and depletion of *A. muciniphila* in patients with NASH-cirrhosis. Behary et al. (2021) identified enriched species composition in patients with NAFLD-HCC, including *Bacteroides cecum, Ruminococcus gnavus, Veillonella parvula, Bacteroides xylanisolvens*, and *Clostridium bolteae*, that may promote tumorigenesis via the Toll-like receptor 4 (TLR4)/nuclear factor kappa-B (NF-κB) pathway. These changes are summarized in Figure 2.

Virome studies further reveal changes in enterovirus composition in patients with NAFLD. A cross-sectional study of 73 patients with NAFLD found reduced numbers of *Lactococcus* and *Lactobacillus* phages and an increased number of phiAT3 phages (Lang et al., 2020). In addition, Chen et al. (2016) reported that viral diversity differed significantly between patients with NAFLD, different disease stages, and study and control groups and that the presence of enterovirus was correlated with NAFLD severity. Figure 2 highlights the unique microbial profiles associated with the different stages of liver disease, emphasizing the potential role of the gut microbiome in disease progression.

Currently available non-invasive diagnostic tools struggle to distinguish NAFLD stages, leading to ambiguous links between microbiota and pathology. This ambiguity is compounded by the fact that most studies have focused on bacterial groups, with limited attention to fungi and viruses. Small sample sizes and lack of multicentre validation further limit generalizability.

Future research should pursue a multimodal diagnostic platform integrating liver spatial transcriptomics with fecal virome sequencing. Developing new biomarker combinations could improve diagnostic accuracy. Understanding the proinflammatory synergy between fungi and bacteria in patients with NASH is also essential. Macrogenomic co-occurrence analysis may clarify these interactions. Monitoring phage dynamics in high-risk patients with NAFLD-HCC may offer early warning markers for timely intervention.

### 3.2 Biological factors affecting heterogeneity in NAFLD gut microbiota studies

#### 3.2.1 Lifestyle

Diet strongly influences gut microbiota. High-fat and highly processed foods can induce dysbiosis, which is closely linked to NAFLD onset and progression (Pi et al., 2024). Variability in dietary habits across populations contributes to heterogeneity in microbiota results. This section examines the effects of alcohol, nicotine, substance use, and circadian rhythms on the NAFLD-microbiota relationship.

#### 3.2.2 Alcohol

The relationship between alcohol and NAFLD is complex. Although NAFLD excludes excessive alcohol consumption by definition, the impact of light-to-moderate alcohol consumption on NAFLD remains a focus of research (Hashimoto et al., 2015; Wongtrakul et al., 2021). Most studies suggest that low-to-moderate alcohol consumption may reduce NAFLD risk and progression (Hamaguchi and Kojima, 2013). For example, Ajmera et al. (2018) reported less improvement in steatosis and NASH among moderate drinkers than in abstainers. A meta-analysis of 8,936 participants showed a lower risk of advanced liver fibrosis in moderate drinkers with NAFLD (Wijarnpreecha et al., 2021). However, the findings are inconsistent. A study of 132 bariatric surgery patients found no association between alcohol intake and histological severity (Cotrim et al., 2009). This inconsistency may be related to small sample size and unadjusted confounders such as genetics, diet, and lifestyle.

Alcohol also affects gut microbiota. Low-to-moderate alcohol consumption can shift gut microbial communities, including those that produce SCFAS (Morrison and Preston, 2016). Caslin et al. (2019) reported higher *A. muciniphila* levels among moderate alcohol consumers, reducing the severity of experimental autoimmune encephalomyelitis. Alcohol may also disrupt gut barrier integrity and gut–brain signaling (de Timary et al., 2015). Moreover, alcohol influences the gut virome. Hsu et al. (2023) reported depletion of *Lactobacillus* phages and increased bIL67 in patients with NAFLD with moderate alcohol consumption compared to those with no or low consumption of alcohol.

These findings suggest that alcohol influences NAFLD via multiple microbiota-related pathways, although the mechanisms remain unclear. Current studies have focused on low-to-moderate alcohol intake, with limited data on heavy drinking. Future large-scale longitudinal studies should quantify alcohol consumption gradients and correlate them with microbial ethanol production through metabolomics to define thresholds for alcohol-induced hepatotoxicity.

#### 3.2.3 Nicotine

Nicotine, the primary component of cigarettes, has a strong influence on gut microbiota composition and NAFLD progression. Smoking is strongly associated with oral and gut dysbiosis, marked by increased *Prevotella* abundance and reduced microbial diversity (Wu et al., 2016). Mechanistically, nicotine synergizes with a high-fat diet to exacerbate hepatic and muscular steatosis, potentially via enhanced abdominal lipolysis and altered lipid metabolism (Sinha-Hikim et al., 2014). In smokers, the gut microbiota shifts include elevated *Prevotella*, decreased *Bacteroidetes*, and lower Shannon diversity—partially reversible after cessation (Stewart et al., 2018). Smoking also increases intestinal permeability and alters luminal pH, promoting pathogenic colonization (Chen J. et al., 2024; Su et al., 2025) and disrupting the GLA, leading to hepatic lipid buildup and insulin resistance (Miao et al., 2023; Sun and Debosch, 2023).

Emerging evidence links electronic cigarettes to microbiota changes, with increased *Prevotella* abundance and depletion of *Anaerobacter*, despite stable overall diversity (Stewart et al., 2018). A 31-year study found a 99% higher NAFLD risk in individuals who were persistently exposed to second-hand smoke; further, heavy smoking (>10 pack-years) has been correlated with higher all-cause mortality in women (Liu et al., 2013; Charatcharoenwitthaya et al., 2020; Wu F. et al., 2021). *Bacteroides xylanisolvens* may mitigate NAFLD by reducing intestinal nicotine bioavailability (Chen et al., 2022).

These findings highlight the pathogenic link between nicotine exposure, microbiota dysregulation, and NAFLD, although the mechanisms remain unclear. Preclinical studies suggest the therapeutic potential of probiotics such as *B. xylanisolvens* in countering nicotine-associated NAFLD (Lee et al., 2018), but randomized controlled trials and mechanistic animal studies are needed. Current research is limited by poor quantification of nicotine exposure, confounding by other tobacco toxins, and reliance on cross-sectional designs, which hinder causal inference.

Emerging nicotine delivery systems, particularly electronic cigarettes, are underexplored. Most studies on vaping involve small, homogeneous cohorts (*n* < 100) and short follow-up periods (< 6 months), yielding inconsistent results. Future research should prioritize longitudinal studies assessing the effects of smoking cessation or nicotine replacement therapies on the gut microbiota and metabolic status of patients with NAFLD. Additionally, screening and validating engineered strains that degrade nicotine in animal models could offer new therapeutic avenues.

#### 3.2.4 Drugs

Pharmacotherapy is a major source of interindividual heterogeneity in gut microbiota composition among patients with NAFLD (Li et al., 2025), driven by bidirectional drug-microbiota interactions that affect both therapeutic outcomes and microbial ecology (Chen L. P. et al., 2024; Liu et al., 2024). Metformin illustrates this complexity: approximately 30% of users experience gastrointestinal side effects linked to *Escherichia coli* overgrowth (Sun et al., 2018; Mueller et al., 2021), and its strain-specific effects on gut microbes have varied across studies (Rosell-Díaz and Fernández-Real, 2024; Szymczak-Pajor et al., 2025). For example, *Bacteroidetes* abundance shows conflicting trends depending on host metabolic status (Ejtahed et al., 2019); meanwhile, increments in *Blautia* and *Butyrivibrio* coupled with a depletion in *Faecalibacterium* is a signature pattern associated with insulin sensitivity (de la Cuesta-Zuluaga et al., 2017; Barengolts et al., 2018; Pastor-Villaescusa et al., 2021). Microbiota responses to metformin differ markedly among patients with NAFLD, reflecting differences in study design, participant characteristics, analytical methods, and individualized nature of drug–microbiota interactions (Jang et al., 2024).

Cardiovascular drugs also modulate the microbiota in NAFLD. SGLT2 inhibitors promote hepatoprotection through *Akkermansia*-enriched remodeling, while thiazolidinediones and DPP-4 inhibitors have neutral or harmful effects on hepatic steatosis (Zhang S. et al., 2024). Statins alter gut microbial communities by inhibiting 3-hydroxy-3-methylglutaryl coenzyme A reductase, potentially explaining the lower T2DM incidence in obese users (5.9% vs. 17.7%) (Zhou H. et al., 2023; Vieira-Silva et al., 2020). Proton pump inhibitors cause long-lasting dysbiosis, marked by *Enterobacteriaceae*/*Lactobacillaceae* abundance and *Ruminococcaceae*/*Bifidobacteriaceae* depletion (Jackson et al., 2016). These changes are linked to NAFLD progression and persist for over 2 years post-treatment (Imhann et al., 2016). Even brief antibiotic exposure (< 7 days) causes long-term microbial diversity loss and functional gene depletion, promoting pathobiont overgrowth and hepatic inflammation (Jernberg et al., 2010). In Finnish children, initial macrolides use led to depletion of *Actinobacteriaceae* and an increase of *Anaplasma* and *Aspergillus* phyla (Korpela et al., 2016).

Although some studies considered drug-related variables, most have focused on single drugs, neglecting the impact of polypharmacy effects and their synergistic or antagonistic effects on microbial metabolism. Notably, microbial responses to the same drug varied widely between individuals. Longitudinal data on microbiota resilience and antibiotic resistance gene enrichment in patients with NAFLD are lacking. Future studies should assess the long-term effects of various drugs on the gut microbiome and NAFLD and develop standardized criteria for assessing drug use, including information on the duration of drug use, dosage, and co-administration, to improve the comparability of results. In particular, multicentre, large-sample clinical studies are needed to validate the prevalence of drug-microbiome interactions.

#### 3.2.5 Circadian rhythm deviation

Circadian misalignment is a key modulator of hepatic pathophysiology, with bidirectional links between chronobiological disturbances and metabolic dysregulation in NAFLD (Bolshette et al., 2023). The liver, which governs ~43% of rhythmic transcriptomes (Zhang et al., 2014), depends on circadian regulation of lipid and glucose metabolism via signaling from the suprachiasmatic nucleus to peripheral clocks (Panasiuk et al., 2024; Speksnijder et al., 2024; Petrenko et al., 2020). Modern lifestyles increasingly disrupt circadian rhythms, contributing to metabolic diseases (Cui et al., 2024) and gut microbiota dysbiosis. The microbiota, considered a “second circadian pacemaker”, shows diurnal oscillations in B/F ratios and microbial metabolite production (Lal et al., 2024; Adafer et al., 2020).

Time-restricted feeding, a chrononutrition strategy, has shown promise in restoring microbial rhythmicity (e.g., *Lactobacillus, Mucispirillum*, and acetate-producing taxa) and reducing hepatic steatosis in preclinical animal models (Schrader et al., 2024; Snijder and Axmann, 2019; Xia et al., 2023). However, current research is largely preclinical, lacks large-scale human data, and offers limited insight into the GLA. To our knowledge, no study has established a framework for personalized circadian-based interventions.

Future studies should employ multi-omics approaches (e.g., transcriptomics and metabolomics) to map circadian-microbial networks and clarify clock-controlled pathways, such as REV-ERBα-mediated bile acid regulation, as potential therapeutic targets. Large prospective cohort studies are needed to build predictive models linking circadian disruption to metabolic disease risk. In summary, circadian rhythm research offers new insights into liver metabolism and may inform targeted prevention and treatment strategies. With further advances, personalized chronotherapeutic approaches are poised to become a key area in future medicine.

### 3.3 Genetic factors affecting heterogeneity in NAFLD gut microbiota studies

Genetic susceptibility plays a central role in NAFLD pathogenesis, influencing both hepatic metabolism and gut microbiota architecture. However, over 90% of microbiome studies in NAFLD have not included host genetic data, and fewer than 15% have adjusted for key confounders such as ancestry, epigenetic modifiers, or familial clustering (Monga Kravetz et al., 2020; Kabbani et al., 2022).

The *PNPLA3 I148M* variant, the strongest genetic risk factor for NAFLD progression, illustrates gene-microbiota crosstalk. Carriers show 2.3-fold higher intestinal permeability, enrichment of *Mycobacterium* spp., and increased circulating endotoxins, which activate hepatic inflammation via TLR4/NF-κB signaling, than non-carriers (Park et al., 2024; Qin et al., 2022). Multi-omics frameworks integrating genome-wide association studies, metagenomics, and metabolomics reveal that genetic variants (e.g., *TM6SF2* E167K and *MBOAT7* rs641738) influence microbial bile acid metabolism and butyrate synthesis, both key drivers of hepatic lipotoxicity (Sehgal et al., 2022; Huang et al., 2023a; Tacke et al., 2023).

Future research should prioritize large, multi-ethnic cohort studies to validate the impact of genetic variations on NAFLD and the gut microbiome and support personalized therapies. Multi-omics technologies and longitudinal studies are needed to unravel the mechanisms underlying gene-microbiota interactions.

### 3.4 Dysfunction of the GLA

Recent population-based cohort studies confirm that microbial dysbiosis and metabolic dysfunction jointly drive NAFLD progression (Takayama et al., 2021). As the core of enterohepatic circulation, imbalance of the GLA is a key pathological factor underlying NAFLD heterogeneity and microbial community shifts (Mignini et al., 2024; Aron-Wisnewsky et al., 2020).

Under normal conditions, the intestinal epithelium acts as a selective barrier via tight junction proteins (e.g., occludin and zonula occludens-1), preventing microbial translocation into the portal circulation (Di Vincenzo et al., 2024) (Figure 2). However, insulin resistance and oxidative stress disrupt the integrity of this barrier, increasing permeability and enabling bacterial migration to the liver via the portal vein. This translocation triggers hepatic inflammation and promotes dysbiosis through local microenvironmental changes (Zhou et al., 2024).

**Figure 3:**
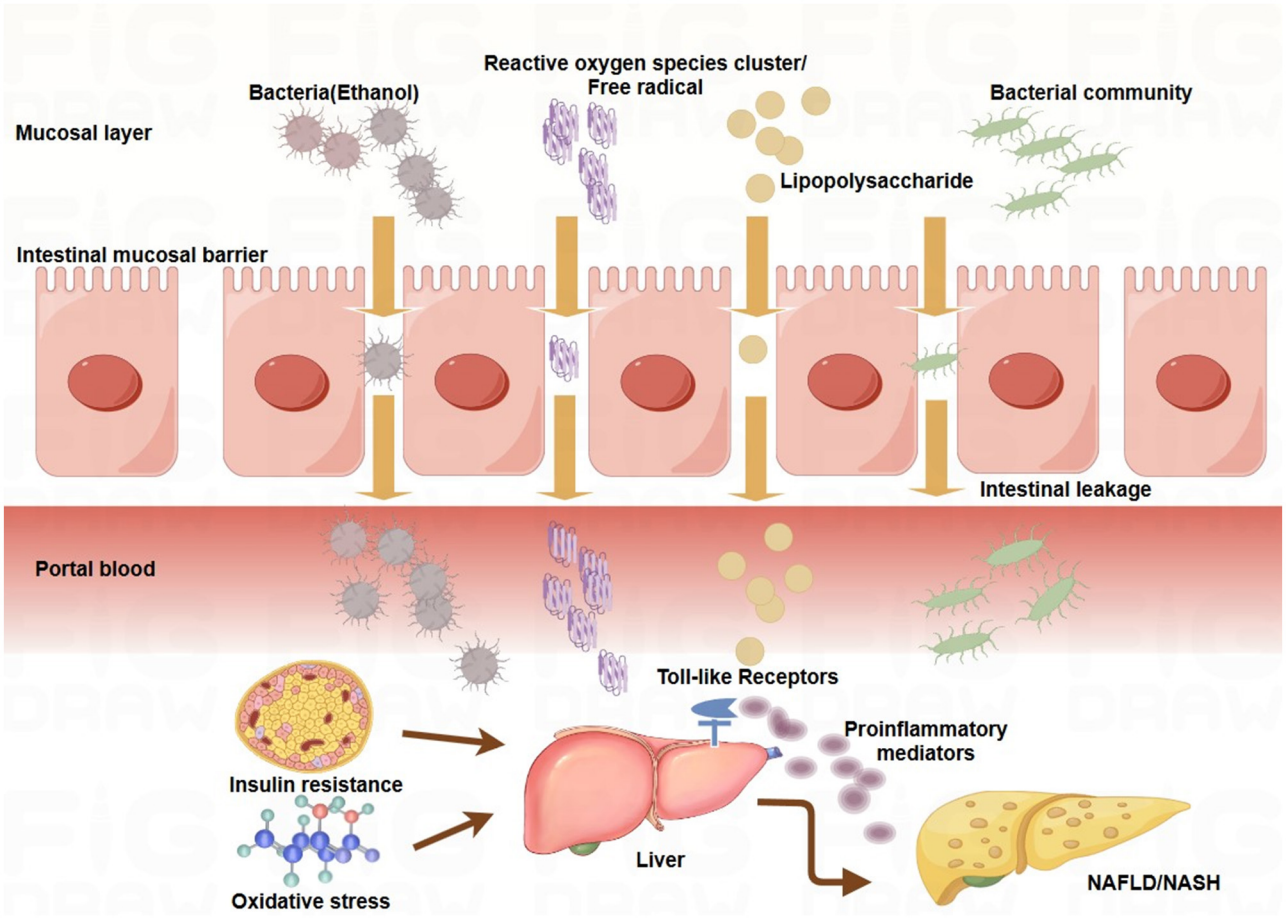
Mechanisms underlying the gut-liver axis in NAFLD/NASH. The intestinal epithelial barrier is compromised under pathological conditions such as insulin resistance and oxidative stress, increasing mucosal permeability. This allows translocation of bacterial components, such as lipopolysaccharide (LPS), and metabolites, such as bacterial ethanol, into the portal circulation. In the liver, these products activate toll-like receptors (TLRs) on hepatic cells, triggering the release of proinflammatory cytokines, hepatic inflammation, and lipid accumulation. These processes contribute to the development and progression of nonalcoholic fatty liver disease (NAFLD) and nonalcoholic steatohepatitis (NASH). Key pathways linking gut microbiota to NAFLD/NASH: Endotoxemia: LPS derived from Gram-negative bacteria activates TLR4 signaling in hepatic cells, promoting proinflammatory cytokine production and hepatic inflammation. Bacterial ethanol: Gut-derived ethanol increased oxidative stress by generating reactive oxygen species (ROS), damaging hepatic cells, and exacerbating inflammation. Bile acid metabolism: Gut microbiota dysbiosis disrupts bile acid metabolism, promoting insulin resistance, a key factor in the pathogenesis of NAFLD/NASH. Energy harvest: The fermentation of dietary fibers by the gut microbiota leads to the production of short-chain fatty acids (SCFAs) that regulate lipid metabolism. SCFA imbalance contributes to hepatic steatosis and inflammation.

Large cohort data link elevated LPS levels in the portal system with increased TLR4 activation, initiating a proinflammatory cascade via the NF-κB pathway, a mechanism that is commonly observed in patients with NAFLD (Smith et al., 2020; Lu et al., 2024; Wu et al., 2024) (Figure 3). Metabolomics studies have shown that gut microbes influence hepatic lipid metabolism and inflammation by altering bile acid composition (reduced primary/secondary bile acid ratio), increasing endogenous ethanol, and decreasing SCFA production (Takayama et al., 2021; Wang et al., 2022). These changes create a feedforward loop through the GLA, perpetuating dysbiosis. For example, altered bile acid metabolism suppresses *Clostridium* species and favors *Proteobacteria* proliferation, whereas SCFA depletion weakens suppression of pathobionts such as *Enterobacteriaceae* (Wu J. et al., 2021; Samy et al., 2024; Xu et al., 2024).

Although serum biomarkers (LPS and zonulin) correlate with NAFLD severity (Guan et al., 2022; Teunis et al., 2022; Wang et al., 2020), most of the evidence is observational, with limited mechanistic studies focusing on GLA pathways. Methodological variability in permeability assays and microbiota profiling also hampers comparability.

Fecal microbiota transplantation has shown promise in restoring GLA balance in early clinical trials, although standardized protocols and long-term outcomes await validation (Abenavoli et al., 2022). Fecal microbiota transplantation has also demonstrated efficacy in attenuating gut microbial heterogeneity, suggesting that microbiota remodeling may mitigate NAFLD progression (Xue et al., 2022). Future research should prioritize (1) large-scale, multi-ethnic cohort studies to identify population-specific variations in gut microbiota composition and function, (2) development of advanced methodologies for assessing the gut barrier, such as artificial intelligent-driven multi-omics platforms, and (3) real-time monitoring using novel paracellular permeability biomarkers (e.g., tight junction protein fragments) such as nanopore biosensors.

Fecal microbiota transplantation optimisation requires rigorous randomized controlled trials to define donor-recipient compatibility, delivery routes (oral vs. colonoscopic), and long-term efficacy. Additionally, scalable formulations, such as lyophilised microbial consortia or synthetic biotic cocktails, are needed to ensure reproducibility and clinical utility.

### 3.5 Methodological factors affecting heterogeneity in NAFLD gut microbiota studies

#### 3.5.1 Study cohort characteristics

Differences in participant age, sex, and geographic region have been found to influence gut microbiota composition in NAFLD research.

##### 3.5.1.1 Age and sex heterogeneity

Age- and sex-related differences in gut microbiota play key roles in influencing NAFLD progression. The microbiota evolves with age, shifting from infancy to adulthood and further changing in old age (Ghosh et al., 2022). Pediatric patients with NAFLD exhibit distinct histological (e.g., portal inflammation without ballooning) (Cross et al., 2024) and microbiota signatures (e.g., *Oscillospira* depletion and *Ruminococcus* enrichment) compared to adults (Shi et al., 2021), suggesting developmental-stage-specific host-microbe interactions (Gancz et al., 2023; Sisk-Hackworth et al., 2023).

Sex hormones also influence microbial communities. Estrogen depletion in postmenopausal women is associated with increased β-glucuronidase activity and altered bile acid metabolism (Chen et al., 2019; Escouto et al., 2023; van Trijp et al., 2021; Hui et al., 2022). Male patients with NAFLD show higher *Dialister* and *Streptococcus* abundance than women, likely due to sex steroid-mediated modulation of bile acid pools, immune response, and epithelial barrier function (Ronan et al., 2021).

Despite these differences, studies on children, adolescents, and the elderly remain limited. Future research should use age-stratified cohorts to clarify microbiota characteristics and their roles in NAFLD/NASH across life stages. Special attention should be given to child and adolescent populations whose gut microbiota are still maturing and whose NASH histology differs from that of adults. Sex-stratified analyses are also needed to explore the influence of hormones, the immune system, and metabolism on microbiota in NAFLD/NASH, particularly in women across puberty, pregnancy, and menopause.

##### 3.5.1.2 Geographic region and dietary habits

Geographic differences in gut microbiota, shaped by diet and lifestyle, are critical determinants of NAFLD pathogenesis. Multinational studies from South America (Brazil) (Escouto et al., 2023), North America (USA) (Trebicka et al., 2024), Asia (China) (Yifu et al., 2022), and Europe (Italy and the Netherlands) (Calabrese et al., 2022; van Trijp et al., 2021) have shown variations in microbial composition linked to ethnicity, culture, and lifestyle (Mehal and Schwabe, 2022). NAFLD prevalence was highest in Hispanic/Latino populations (37.0%), intermediate in non-Hispanic individuals of European ancestry (29.3%), and lowest in non-Hispanic individuals of African ancestry (24.7%) (Huang et al., 2021). Japanese Americans are more susceptible to NAFLD than African Americans (Hullar et al., 2021), often caused by reduced microbial diversity (Zhou et al., 2019).

Diet further modifies NAFLD risk (Shen et al., 2017; Hydes et al., 2020). Low-calorie and low-glycaemic diets reduce hepatic steatosis via microbiota-dependent enhancement of gut barrier integrity (Fernández et al., 2022; Houttu et al., 2021). In contrast, Western diets worsen NAFLD by driving central obesity and inflammation (Bennett et al., 2022). Ethnic dietary patterns influence NAFLD prevalence, as seen in China, where Uyghurs show 2.1-fold higher rates than Han, Kazakh, or Mongolian populations (Zhou et al., 2019).

Geographic and dietary factors jointly shape the gut microbiota and NAFLD/NASH risk. Future studies should use multiregional cohorts with standardized dietary and lifestyle data. Multi-omics approaches can identify region-specific microbial signatures linked to disease progression, particularly in the context of urbanization-driven dysbiosis. Factors such as antibiotic overuse, environmental toxicants, and urban dietary shifts may further disrupt microbiota and gut integrity. Interdisciplinary investigations will bridge critical gaps in the understanding of microbiota-mediated NAFLD pathogenesis across diverse populations, leading to the development of personalized NAFLD prevention strategies tailored to regional and cultural contexts.

#### 3.5.2 Heterogeneity of analytical methods

##### 3.5.2.1 Diagnostic variability as a confounding factor

Diagnostic inconsistency is a source of heterogeneity in gut microbiota profiles across NAFLD studies. Although liver biopsy is the histopathological gold standard, its invasive risks (e.g., hemorrhage and infection), sampling bias, and interobserver variability limit its clinical utility (Cao et al., 2022). Consequently, many studies have adopted non-invasive tools such as ultrasonography, MRI-PDFF, and transient elastography. However, these methods exhibit significant limitations: ultrasonography has low sensitivity for mild steatosis and only detects lesions with >30% hepatic fat (Ezenwuba and Hynes, 2024); MRI-PDFF quantifies fat but does not assess inflammation and fibrosis (Huang et al., 2025). Transient elastography performs poorly in patients with obesity and cannot distinguish simple steatosis from NASH (Gawrieh et al., 2024).

Staging ambiguity further complicates research. Non-invasive techniques often fail to align with histological staging (e.g., steatosis, activity, fibrosis score S0–S4), leading to oversimplified classifications (e.g., “NAFLD” vs. “NASH”) that obscure disease progression (Zoncapè et al., 2024; Barr, 2025). Meta-analyses incorporating studies using different diagnostic criteria increase variability. For example, some studies diagnose NAFLD using ultrasonography (Hui et al., 2022) or elastography (Calabrese et al., 2022), while others rely on biopsy-based steatosis, activity, fibrosis scoring (Yifu et al., 2022; Trebicka et al., 2024; Loomba et al., 2021; Behary et al., 2021), fibrosis staging (Ponziani et al., 2019; Loomba et al., 2017), or NASH/NAFLD distinctions (Hoyles et al., 2018; Alferink et al., 2021; Zhang et al., 2021; Demir et al., 2022). Such methodological inconsistencies hinder the establishment of consistent microbiota profiles.

To achieve consistent microbiota profiles, a multifaceted approach that integrates standardized protocols, advanced technologies, and biomarker discovery is essential. Phase-specific diagnostic guidelines should align diagnostic tools with disease stage. The FIB-4 index combined with ultrasonography is cost-effective for early screening (Schwabe et al., 2020); MRI-PDFF paired with elastography enhances fat quantification and fibrosis assessment in the mid-to-late stages; liver biopsy should be reserved for diagnostically challenging cases for optimizing the balance between cost, risk, and accuracy (Arrese et al., 2021). Multimodal diagnostic platforms must integrate imaging, histology, blood biomarkers (e.g., gut-derived LPS and secondary bile acids), and microbiota data to enable machine learning-driven, non-invasive staging.

Precision biopsy cohorts should simultaneously analyse gut microbiota, hepatic immune microenvironments, and circulating metabolites in patients with clear pathological biopsy-based staging (e.g., steatosis, activity, fibrosis scores). Spatial transcriptomics can map microbe-associated molecular patterns in inflamed or fibrotic liver regions, revealing pathological changes through the GLA. Future research should also focus on novel noninvasive biomarkers such as endotoxin-producing gram-negative bacteria, fecal virulence markers, fungal markers, or blood metabolite ratios (e.g., branched-chain amino acid/aromatic amino acid ratios) to develop diagnostic indices (Han et al., 2023). Detection of microbial DNA fragments in exosomes or liver-specific cfDNA methylation may further indicate gut-liver interactions.

##### 3.5.2.2 Heterogeneity in sequencing methodologies

Sequencing methods affect the comparability of the gut microbiome findings of large cohort studies. For instance, 16S rRNA sequencing in one cohort identified taxa linked to NAFLD, whereas metagenomic sequencing approaches with broader gene coverage yielded non-overlapping results (Xu et al., 2023). Metagenomics captures gene-level data but cannot distinguish viable from dead microbes and is prone to DNA loss, contamination, or degradation (Zhou K. et al., 2023).

Pre-analytical variables, such as fecal collection methods, freezing delays, and DNA extraction protocols, can influence microbial profiles (Zeng et al., 2024). Questionnaire-derived variables (e.g., defecation frequency, diet) add bias due to inconsistent collection and standardization across cohorts, complicating interpretation and generalization (Park et al., 2021).

Future studies should combine 16S rRNA sequencing, shotgun metagenomics, and single-cell sequencing to achieve high-throughput profiling, strain-level resolution, and functional potential analysis. For example, Ke et al. (2019) combined metaproteomics (protein expression), metabolomics (metabolite dynamics), and 16S sequencing to show how probiotics modulate diet-induced dysbiosis and improve metabolic outcomes in obese mice. Efforts are also needed to expand virome and archaeome profiling using viral enrichment and methanogen-specific primers (e.g., the *mcrA* gene) and assess their roles in microbial dynamics and methane production, both relevant to NAFLD. Standardizing pre-analytical workflows is essential for reducing technical variability.

#### 3.5.3 Statistical limitations

In NAFLD research, given the ethical concerns over liver biopsy restricting histological data collection (Di Pasqua et al., 2022; Panday et al., 2022; Wang et al., 2024), many studies rely on non-invasive diagnostics with known variability. Some studies have used non-invasive assays (e.g., MRI-PDFF) to report differences in microbial abundance with nominal *P*-values, overlooking multiple testing burdens. For example, a study linking *Bacteroides* abundance to BMI in obese children (Morán-Ramos et al., 2022) lost significance after false discovery rate correction, a mandatory practice in genomics, yet inconsistently applied in microbiome research.

Future studies must adopt false discovery rate correction (e.g., Benjamini–Hochberg procedures) to reduce false positives and ensure statistical rigor. Bayesian hierarchical models with shrinkage priors (e.g., horseshoe priors) can address sparse signals and population stratification. Machine learning methods, such as elastic net regression, are suitable for multicollinear microbial data. Power analysis tools (e.g., *micropower* R package) should guide sample size planning, and active learning frameworks should be used to prioritize the enrolment of phenotypically extreme cases (e.g., patients with discordant imaging-histologic staging).

Given NAFLD's potential for reclassification and temporal microbiome shifts, hybrid designs using Mendelian randomization (e.g., *PNPLA3* rs738409 as an instrument) and longitudinal mixed-effects models are needed to establish causality and translate statistical rigor into biologically meaningful insights.

### 3.6 Microbial community dynamics

Dynamic shifts in gut microbiota contribute to interpatient variability in NAFLD. Dysbiosis is bidirectional, driven by host genetics, high-sugar/high-fat diets, or antibiotic use, and worsens with NAFLD progression (Benedé-Ubieto et al., 2024; Di Vincenzo et al., 2024). Despite functional overlap in key metabolic pathways, such as bile acid metabolism and SCFA synthesis, strain-level differences lead to metabolite variability, limiting the utility of single-species analyses (Lau et al., 2024). Microbial communities also adapt rapidly to host conditions, further increasing study heterogeneity.

Future research should use multi-omics methods to capture functional changes in microbial communities. Longitudinal cohort studies using time-series analyses can track microbial trajectories from simple steatosis to NASH, supporting early diagnosis and intervention. Host-microbe interactions, especially the impact of microbial metabolites on hepatic inflammation and lipid deposition, should be investigated using *in vitro* co-culture models and animal experiments. Personalized strategies, including targeted nutrition and tailored probiotics/prebiotics, should also be explored based on individual microbiome dynamics.

### 3.7 Interplay between heterogeneity drivers: toward a systems-level understanding

The clinical, biological, methodological, and microbial factors discussed above do not operate in isolation but interact in complex, synergistic ways that fundamentally amplify heterogeneity and challenge simplistic interpretations. For instance, an individual's genetic background (e.g., carriage of the *PNPLA3 I148M* variant) can modulate their metabolic response to drugs like metformin, which in turn induces distinct shifts in the gut microbiota composition and function. Similarly, dietary habits (a biological factor) are deeply entangled with geographic and cultural contexts (a methodological cohort characteristic), creating unique microenvironmental pressures that are conducive to divergent microbial communities. Furthermore, disruptions to the circadian rhythm can exacerbate dysbiosis induced by a high-fat diet, while also altering host drug metabolism, creating a vicious cycle that promotes NAFLD progression through multiple, interconnected pathways.

The current practice of analyzing these factors in isolation, while necessary for initial characterization, limits a holistic understanding. This challenge is compounded by the pervasive methodological heterogeneity across studies, which includes variations in diagnostic criteria (e.g., ultrasound vs. biopsy), sequencing technologies, and statistical approaches. These methodological differences not only contribute directly to observed discrepancies but also confound our ability to disentangle the complex biological interactions described above.

Future research must therefore prioritize integrative analytical frameworks that address both biological and methodological complexity, e.g., frameworks employing interaction terms in multivariate models within large, diverse cohorts to statistically quantify these interdependencies, and frameworks applying multi-omics integration (e.g., merging genomics, metabolomics, and metagenomics) to elucidate the underlying biological networks. Concurrently, the adoption of standardized, phase-appropriate diagnostic pathways, rigorous statistical corrections for multiple testing, and prospective multicentre designs are essential to reduce noise and enhance the comparability of future datasets.

A systems-level approach that captures the dynamic crosstalk between host genetics, lifestyle, environment, and microbial ecology—while minimizing methodological confounders—is paramount for advancing beyond correlation and toward causal, mechanistic insights. This refined understanding is essential for developing personalized microbiota-targeted interventions that account for the unique confluence of factors in each patient.

## 4 Conclusion

This review delineates the intricate relationship between gut microbiome alterations and NAFLD progression while critically addressing the methodological and conceptual limitations that hinder consensus in current research. The key challenges include the predominance of cross-sectional studies obscuring causal links; methodological inconsistencies (e.g., diagnostic criteria, sequencing platforms); and overemphasis on bacterial communities at the expense of fungi, archaea, and virome components. Geographic and demographic biases further limit the generalizability of findings.

To translate these insights into clinical practice, a concerted effort toward implementing integrated translational frameworks is paramount. Specifically, we propose a structured implementation pathway centered on multi-omics integration and precision microbiota therapy:

For multi-omics integration, a logical technical workflow should be adopted: (1) utilizing metagenomics to define taxonomic and functional potential; (2) applying metabolomics (e.g., mass spectrometry) to profile key microbial and host metabolites (e.g., short-chain fatty acids, bile acids, endogenous ethanol); and (3) employing proteomics or host transcriptomics to assess the resultant hepatic inflammatory and metabolic responses. The critical next step is data integration using bioinformatics platforms (e.g., MixOmics, QIIME 2 plugins) and machine learning algorithms (e.g., random forest, neural networks) to identify robust, cross-validated biomarker panels that can stratify patients and predict disease progression.

For precision microbiota therapy, future clinical trials must incorporate several key design elements to ensure validity and clinical relevance: (a) Precisely defined patient cohorts, stratified not just by NAFLD severity but also by baseline microbial signature (e.g., enterotype), genetic risk factors (e.g., PNPLA3 genotype), and major metabolic comorbidity status; (b) standardized and quality-controlled interventions, whether using defined consortia of beneficial strains, engineered probiotics with specific functions (e.g., bile acid metabolism), or rigorously screened fecal microbiota transplantation; (c) clinically meaningful endpoints beyond microbial shifts, primarily focusing on improvements in liver fat content (quantified by MRI-PDFF), histologic activity (for trials including biopsy), and non-invasive fibrosis markers (e.g., ELF test, VCTE); and (d) long-term follow-up schedules (e.g., 1–2 years) to monitor the sustainability of microbial remodeling, long-term safety, and hard clinical outcomes.

Beyond these core strategies, future studies must also prioritize longitudinal multi-omics approaches to resolve microbial functional dynamics across NAFLD stages; standardized protocols for sample processing and data analysis to reduce technical variability; mechanistic validation using organoid models and gnotobiotic systems to test candidate metabolites (e.g., iso-bile acids) and microbial-host crosstalk; diverse population cohorts incorporating age-stratified, sex-specific, and geographically tailored dietary interventions; and emerging frontiers such as phage-microbe networks and precision probiotics for targeted therapeutic development.

A paradigm shift toward systems-level analysis is imperative, leveraging machine learning to integrate multi-dimensional data for personalized risk prediction while bridging bench discoveries to clinical applications through diet-microbiome co-regulation strategies. Addressing these priorities will not only clarify microbiome-NAFLD causality but also unlock novel diagnostic and therapeutic avenues for this globally prevalent disease.
